# Ethanol to Aromatics on Modified H‐ZSM‐5 Part II: An Unexpected Low Coking

**DOI:** 10.1002/asia.202000961

**Published:** 2020-10-20

**Authors:** Markus Seifert, Mathias S. Marschall, Torsten Gille, Clemens Jonscher, Philipp Royla, Oliver Busse, Wladimir Reschetilowski, Jan J. Weigand

**Affiliations:** ^1^ Faculty of Chemistry and Food Chemistry Technische Universität Dresden 01062 Dresden Germany; ^2^ Faculty of Chemistry and Food Chemistry Technische Universität Dresden 01062 Dresden Germany

**Keywords:** heterogeneous catalysis, kinetics, structure-activity relationships, sustainable chemistry, zeolites

## Abstract

In this study a commercial H‐ZSM‐5 zeolite (Si/Al=11) was post‐synthetically modified by a combined dealumination procedure to adjust its catalytic properties for the selective formation of aromatics from ethanol. The solid‐state properties of original and modified zeolites are determined by structural, textural and acidity analysis. The formation of aromatics and durability of the zeolites were investigated depending on space velocity or contact time in the catalyst bed. In particular, the formation rate and desorption of aromatics from solid‐state surface as well as their tendency to form coke precursors by consecutive build‐up reactions determine the formation of coke. Therefore, the rate of build‐up and finished aromatization by hydride transfer (pre‐determined by the kind, location and geometric arrangement of surface acid sites) and the statistical number of reaction events until final desorption at the specific contact time have to be harmonized to increase aromatics yield and to decrease catalyst decay by coke simultaneously.

## Graphical abstract

**A combination of synthesis, post‐synthesis and reaction technology** to enhance aromatics yield and reduce coking in ethanol conversion on ZSM‐5 zeolite is a challenging and multidisciplinary task. A harmonization of product formation speed and product transport through catalyst grain and bed reveals unexpected resonance conditions explained by carbocation‐based reactions.

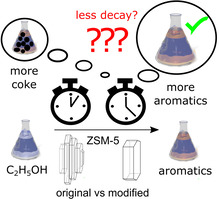

## Introduction

Zeolite H‐ZSM‐5 is a well‐known and frequently used catalytically active material for the conversion of methanol or ethanol to hydrocarbons (MTH and ETH process, respectively) since its invention by Mobil Oil Corporation in 1972.^[1]^ Besides the broad variability in Si/Al ratio from synthesis, post‐synthesis methods help to tune its activity and selectivity, as well as catalyst stability or regeneration. By unrestricted formation of hydrocarbon species during conversion of small alcohols, coke is formed, which blocks the active sites as well as the pores, and therefore causes short‐term deactivation.^[2]^ It must be taken into account here, that not only mass transport and diffusion phenomena within the catalyst grain, but also mass transport processes through the reactor and within the catalyst bed determine catalytic activity, product selectivity and catalyst deactivation.^[3]^ Consequently, the development of a sustainable conversion process from small alcohols, e. g. ethanol, has to be handled as a multidisciplinary task. On the first screening stages of catalyst development,^[4]^ mass transport within the structure of active zeolite H‐ZSM‐5 must be designed from different perspectives:

- Zeolite synthesis, e. g. formation of nano‐crystalline ZSM‐5,^[5]^ determines particle size, morphology and external transport processes.
- Post‐synthesis, e. g. formation of hierarchical structures,^[6]^ helps to reduce transport restrictions of bulky products. In addition, adaption of active site density, e. g. by Si/Al ratio, can reduce the probability of formation of too small or too bulky intermediates.^[7]^
- Finally, catalytic test parameters, e. g. choice of contact time, temperature and pressure, help to optimize the aromatics formation during ethanol conversion.[^7^, ^8^]

The combination of these three aspects is inevitable to understand and control pathways of ethanol to aromatics and the progressive catalyst deactivation by coke formation.

### Scope of this work

Previous investigations revealed scientifically based combinations of dealumination methods to reduce acid site density, to clean pore channels from residual extra‐framework species (EFSPE) and to enhance aromatics yields.[^9^, ^10^] The main goal of this work now is to study short‐term durability behavior of H‐ZSM‐5 zeolites during conversion of ethanol to aromatics by variation of inert gas flow and contact time, respectively. Based on a commercial and modified H‐ZSM‐5 zeolite durability and selectivity trends with varying inert gas flow reveal unexpected distinct product formation parameters for aromatics and coke formation and a decoupling of catalyst decay from coking.

Deeper insight into molecular processes of elementary reactions at active sites in combination with harmonized mass transport result in a new interpretation of preliminary conclusions by da Silva et al.^[7]^ Preferred formation of aromatics at higher contact time is not always coupled to increased deactivation by coke formation.

## Results and Discussion

### I. Solid‐state properties and catalytic performance of commercial and modified H‐ZSM‐5

For description of trends in aromatics formation and catalyst durability during ethanol conversion a commercial H‐ZSM‐5, denoted as original H‐ZSM‐5 (Si/Al=11), and its post‐synthetically modified analogue (Si/Al=20) are compared. The experimental part includes a detailed description of the modification procedure by base, acid, steam and acid treatment.

A short overview of solid‐state and surface properties of original H‐ZSM‐5 in Table 1 reveals some parameters, which are unfavorable for an enhanced production of small aromatics from ethanol conversion. Consequently, catalytic performance in terms of durability and aromatics formation (aromatics index, (***AI***)) offers optimization options. Aromatics index (***AI***) is defined as the ratio of aromatics yield to the theoretical limit from aromatics formation by hydride transfer between small olefins. For further details please consult the supporting information.

**Table 1:** Solid‐state properties of original H‐ZSM‐5 zeolite and modified H‐ZSM‐5 (incl. target values from literature).

H‐ZSM‐5	Original	Modified	Trend or goal
Structural and surface chemical properties			
Si/Al^[a]^	11	20	20–25
Acidity^[b]^ [mmol/g]	0.56	0.41	–
Crystallinity (XRD, long range) [%]	100 (p.D.)	123	–

Textural properties			
BET surface^[c]^ [m^2^/g]	390	410	400
BJH surface^[c]^ [m^2^/g]	61	89	0 (low)
Particle size (D50) [μm]	<12	<7	1–10

Durability			
Coke (10 h ToS) [wt. %]	6.2	3.0	0.0
(TOS)_S_^[d]^ [h]	9	80	– (high)

Selectivity			
(***AI***)^[e]^ [%]	8	23	100

[a] Zeolite dissolution in HF and ICP‐OES analytics, for a description of the goal, see supporting information (Table S1); [b] Temperature‐programmed desorption of ammonia, high‐temperature peak; [c] Physisorption of N_2_, BET and BJH model for micro‐ and mesopore surface; [d] (ToS)_S_ as model parameter to time on stream at 50% deactivation (see experimental part); [e] Aromatics index (***AI***) to quantify the formation of small aromatics compared to the theoretical limit (see experimental part)

Because of high amounts of framework aluminum and high concentration of acidic sites, coke formation is preferred, which leads to a high deactivation constant compared to the underlying deactivation model. Further details are described in the experimental part. Low (***AI***) shows pore narrowing due to EFSPE. Nevertheless, complete regeneration of the catalytically active material by burning of coke until 550 °C in air is possible. The results of the solid‐state and catalytic properties of original H‐ZSM‐5 zeolite after the post‐synthetic modification and the desired catalytic performance achieved for the ethanol conversion to aromatics are summarized in Table 1. It confirms the preparative success. ICP‐OES results as well as (***AI***) indicate an increase of the Si/Al ratio as well as cleaned pores. This is described in more detail in recent work.^[10]^ For ammonia desorption data, powder diffraction patterns and N_2_ physisorption data, please consult the supplementary information, Figure S1–S3.

**Figure 1:**
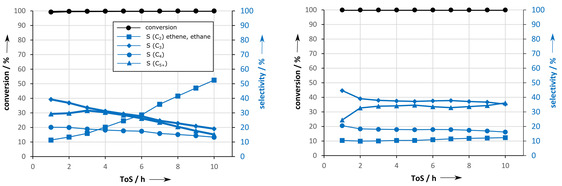
Conversion and product selectivity (C_2_, C_3_, C_4_, C_5+_) depending of the ToS for ethanol conversion on *(left)* original H‐ZSM‐5 and *(right)* modified H‐ZSM‐5.

**Figure 2:**
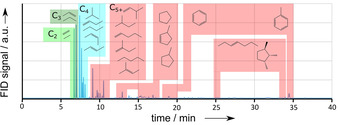
Gas chromatogram of gas phase product spectrum at 3 h ToS for conversion of ethanol at 350 °C on original H‐ZSM‐5, product fractions defined by carbon number in the molecule C_2_, C_3_, C_4_ and C_5+_ are marked.

A comparable look onto the conversion and selectivity plots (Figure 1) reveals almost full ethanol conversion, but increasing C_2_ (mainly ethene) fraction with ToS, which indicates deactivation by coke formation.^[2]^ Meanwhile, selectivities of the other fractions C_3_, C_4_, C_5+_ drop, but their decays differ. A deeper look at the product formation in gas phase is realized by gas chromatography in Figure 2. Consequently, improved catalyst durability and reduced coking are observed with the ability to preserve the regenerability by burning of coke.

**Figure 3:**
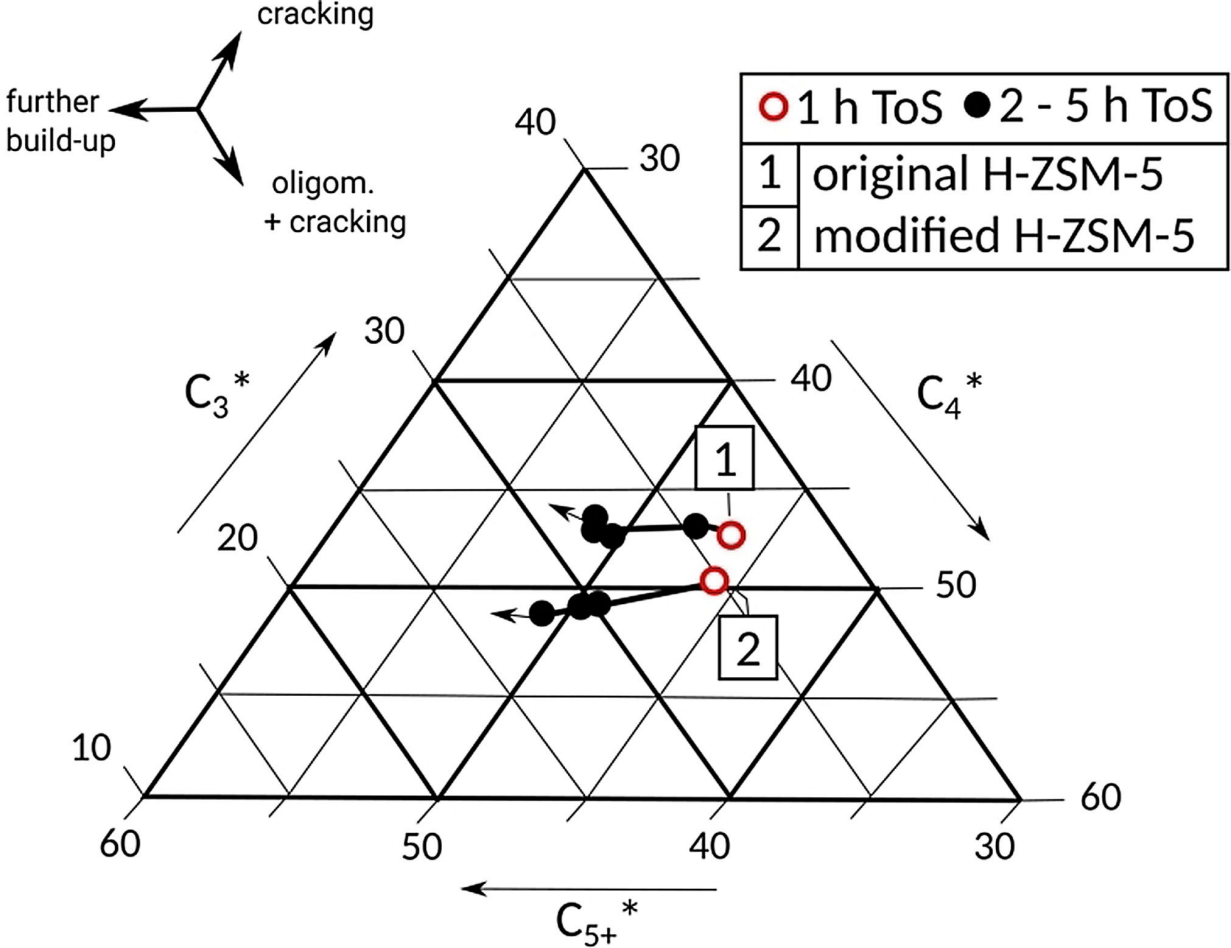
Ternary plot of model‐based formation trends of C_3_, C_4_ and C_5+_ gas‐phase products [wt. %] with ToS [h]: original H‐ZSM‐5 and modified H‐ZSM‐5 during first 5 h ToS.

A ternary plot‐model is illustrated in Figure 3, in which gas‐phase product fractions C_3_, C_4_ and C_5+_ are normalized to current conversion of C_2_. By this normalization a product formation trend becomes visible, which is independent from the fast initial ethanol dehydration process and the ongoing blocking of acid sites by coke (increased C_2_).

Figure 3 shows differences in product formation trends with ToS. C_4_ is mainly attributed to ethene dimerization. Furthermore, butenes (incl. butanes) as C_4_ and propene (incl. propane) as C_3_ indicate cracking processes. Propene and butenes are denoted to the major intermediate olefins, which may leave the pore system after production.^[11]^

Further build‐up reactions lead to bigger molecules C_5+_ in general, which often also may leave the pore system after formation without further growth to coke. Pore cleaning and reduction of active site density by post‐synthetic modification reveals an additional increase in formation of C_4_ products in general and C_5+_ with ToS. This is due to calmed build‐up reactions by avoidance of unrestricted production of bigger coke precursors.

### II. Impact of contact time by variation of nitrogen flow

To minimize the tendency to form coke, a reduction of contact time should help to suppress consecutive reactions towards higher‐weighted coke precursors. By adaption of nitrogen flow between 5–20 L/h (norm.) at constant reaction pressure and weight‐hourly space velocity (WHSV), contact time was modified within the range of 0.8–0.2 s as described in the experimental part (Table 3). It should be noted that higher flow of nitrogen/lower contact time (t<0.2 s) caused overpressure beyond 2.5 bar (abs.) and lower flow/higher contact time (t>0.8 s or no nitrogen) entailed an aggravating transport of ethanol through the reactor.

A simple deactivation model is used, which describes the ongoing coking and deactivation process as mentioned within the ′cigar burn′ model.^[12]^ From this, two parameters, namely the exponential deactivation rate and the extrapolated linear parameter C_2_ in gas phase at start of the reaction are suitable to characterize the deactivation behavior. (see experimental part for further details) A normalization of these parameters to original H‐ZSM‐5 at standard conditions 350 °C and contact time of 0.30 s facilitates the illustration of differences and trends.

**Figure 4:**
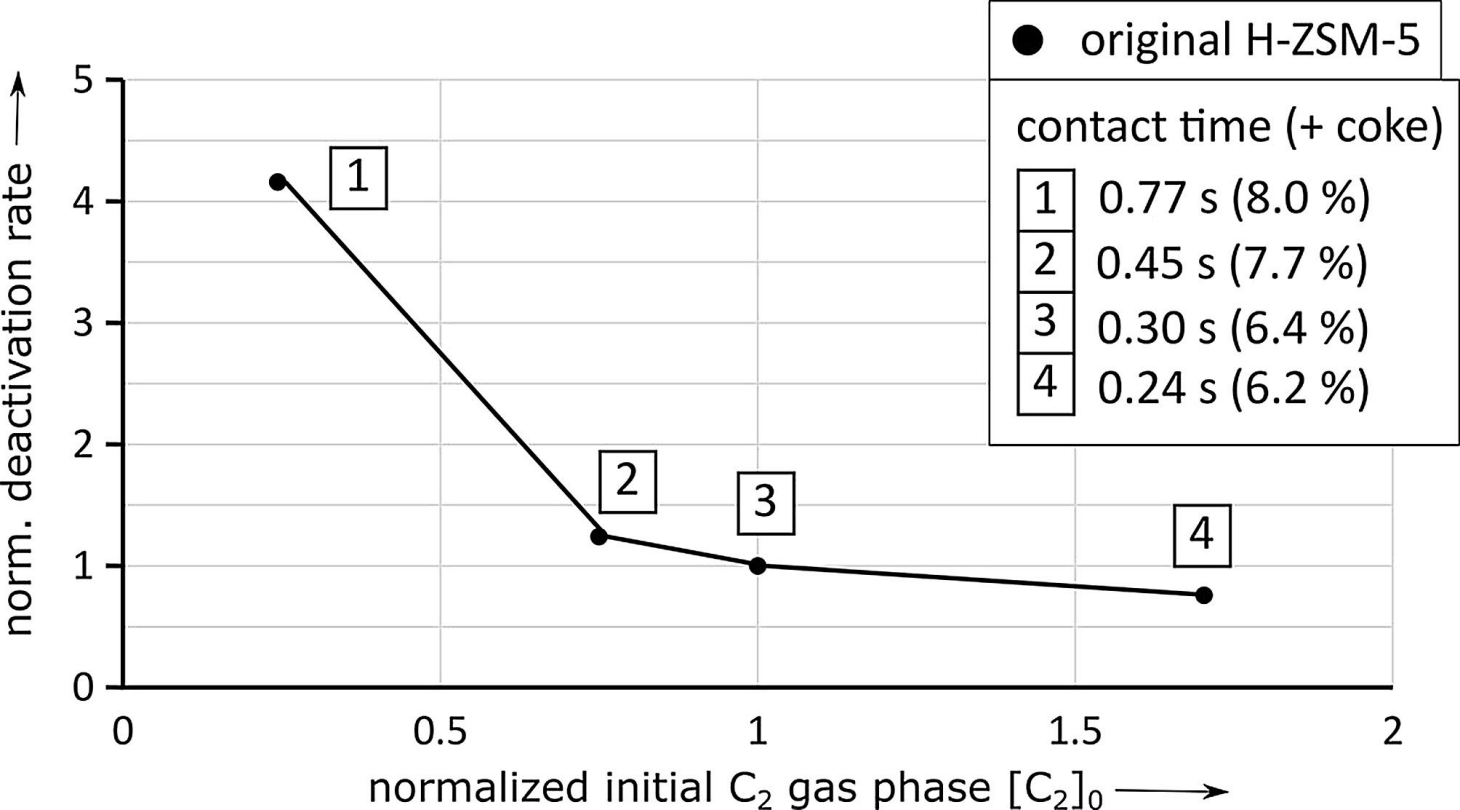
Deactivation and activity plot from underlying kinetic deactivation model as well as the amount of coke in wt. % for original H‐ZSM‐5 after 10 h ToS depending on contact time at constant WHSV=5 h^−1^.

In case of original H‐ZSM‐5 a reduced nitrogen flow increases the contact time (4→1), which increases the amount of coke after 10 h ToS and increases the conversion of C_2_ (Figure 4). In consequence, a consecutive production of C_2_ from ethanol, followed by the formation of higher‐weighted hydrocarbon products from C_2_ as well as coke from higher weighted hydrocarbons seem to be appropriate.

**Figure 5:**
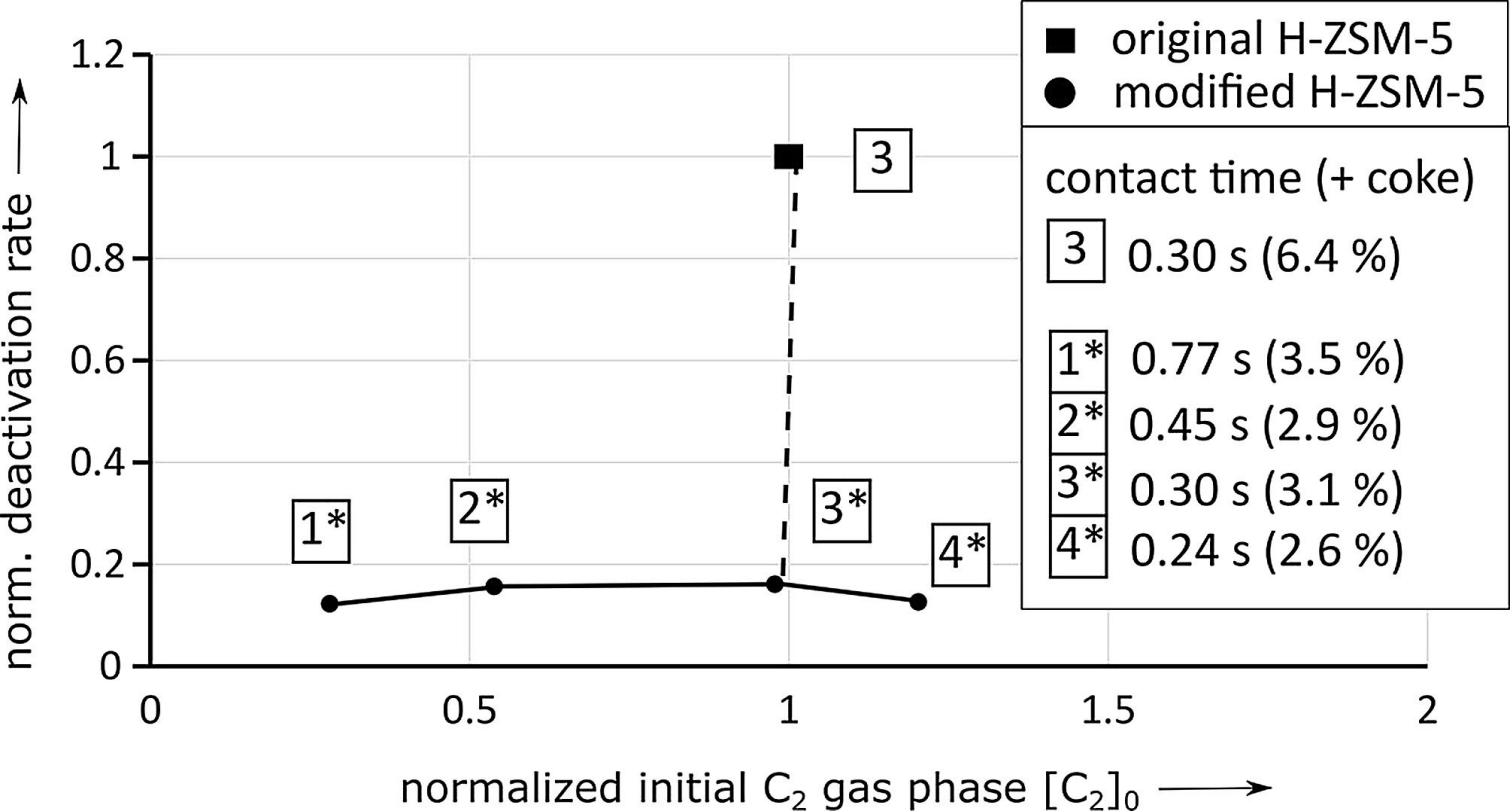
Deactivation and activity plot from underlying kinetic deactivation model as well as the amount of coke in wt. % for modified H‐ZSM‐5 after 10 h ToS depending on contact time at constant WHSV=5 h^−1^.

For modified H‐ZSM‐5, a reduced nitrogen flow (increased contact time 4*→1*) leads again to a reduced conversion of C_2_ towards higher‐weighted hydrocarbons. Meanwhile, reduced amounts of coke and increased deactivation is only observed at contact time <0.3 s (Figure 5). In general, deactivation rate is lower compared to original H‐ZSM‐5, but deactivation by C_2_‐build‐up seems to be decoupled from coke formation within the used parameter set for contact time >0.3 s.

**Figure 6:**
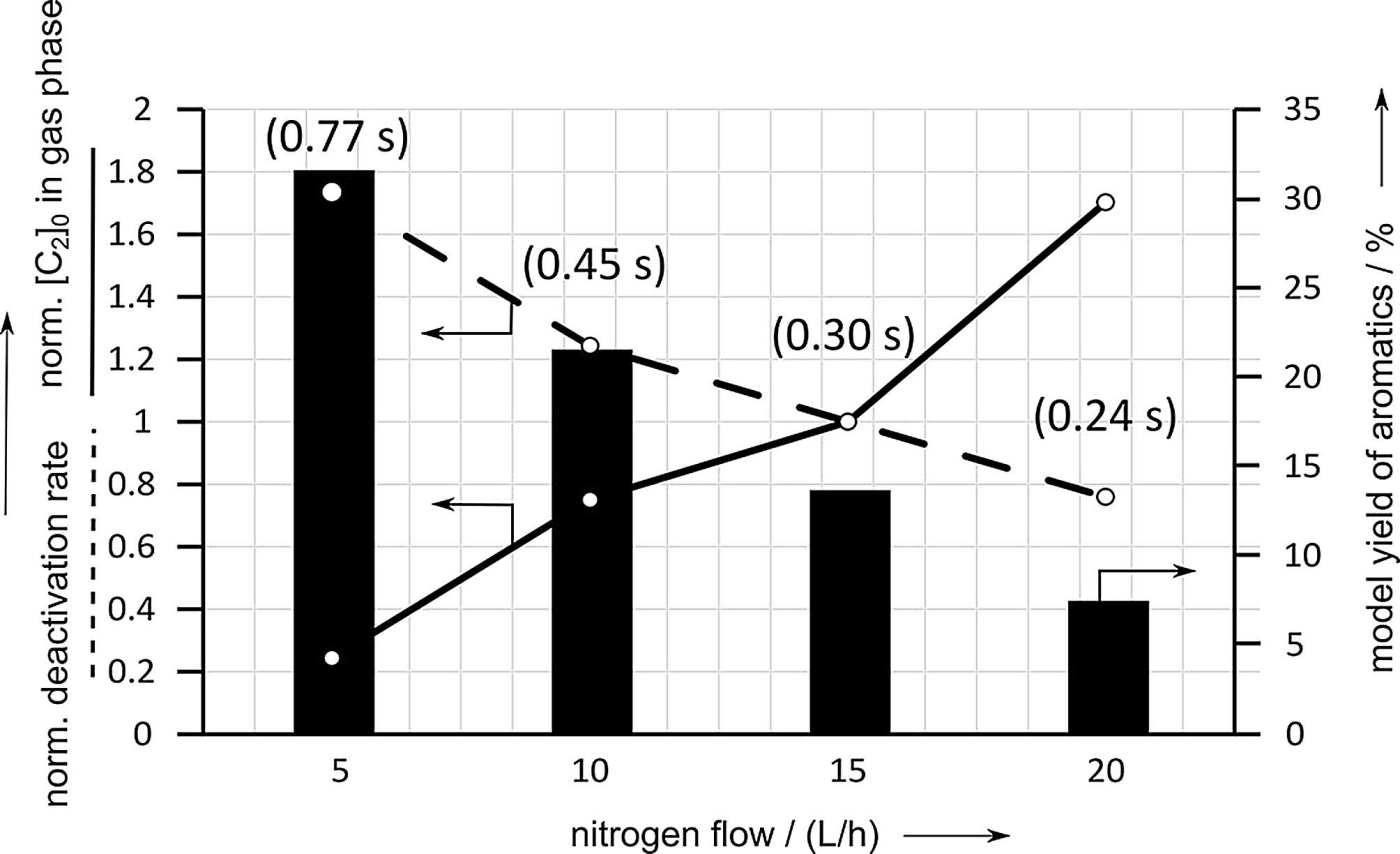
Deactivation and activity plot from underlying kinetic deactivation model as well as the yield of small aromatics after 10 h ToS for original H‐ZSM‐5 depending on contact time at constant WHSV=5 h^−1^.

**Figure 7:**
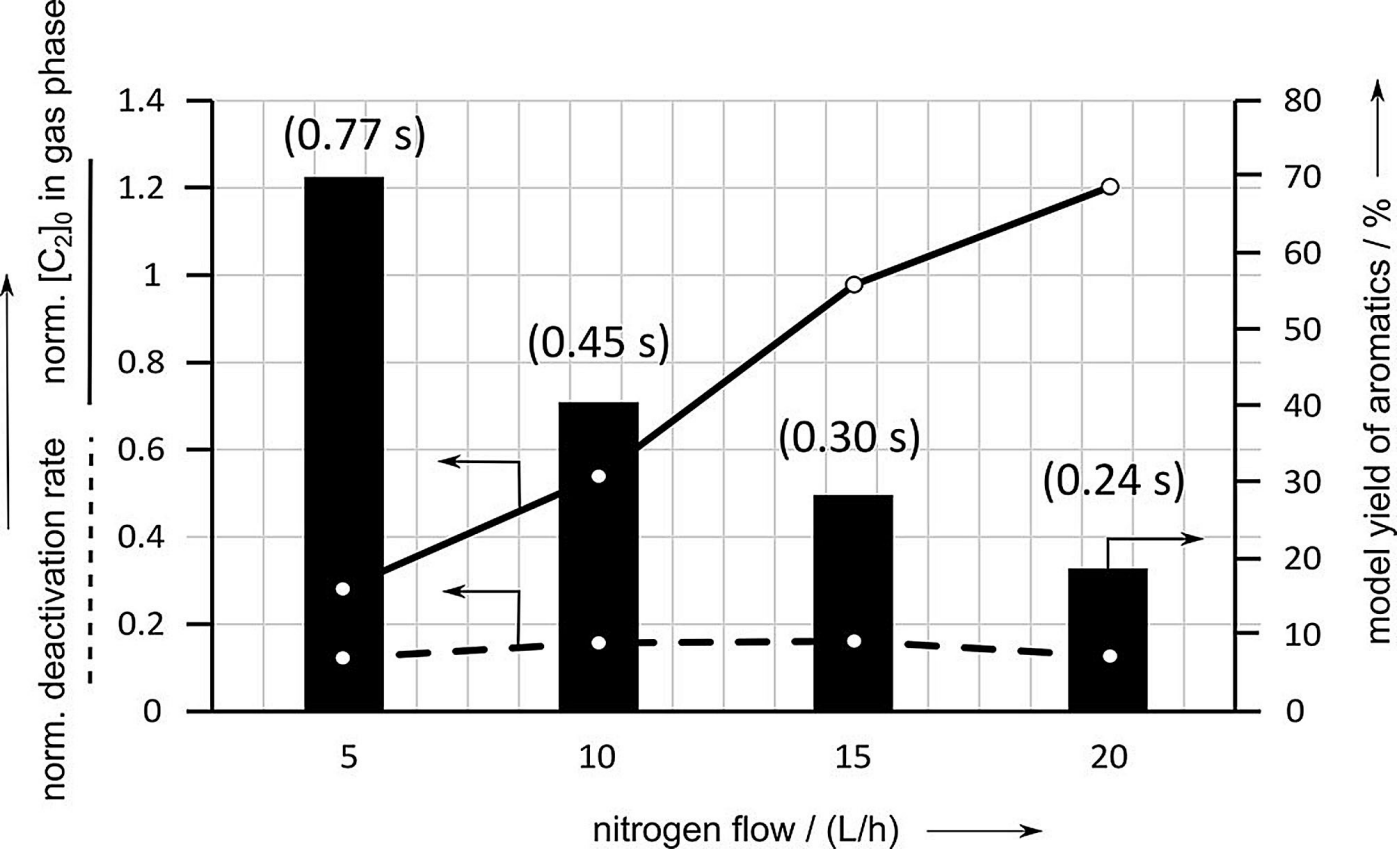
Deactivation and activity plot from underlying kinetic deactivation model as well as the yield of small aromatics after 10 h ToS for modified H‐ZSM‐5 depending on contact time at constant WHSV=5 h^−1^.

As reported by da Silva et al.,^[8]^ a reduced contact time leads not only to decreased amounts of coke (Figure 4 and Figure 5), but also to decreased amounts of small aromatics using both modified and unmodified H‐ZSM‐5 zeolite catalyst (see Figure 6 and Figure 7).

A more detailed comparison of the amount of coke and the amount of monocyclic aromatic products after 10 h ToS reveals in general, there is a preferred formation of small aromatics after combined post‐synthetic treatment at the expense of the coke yield and the yield of small olefins.^[13]^

As the aromatics yield and coke content show similar trends towards contact time by variation of nitrogen flow, catalyst decay seems to be in a kind of resonant parameter regime with a maximum or a plateau after post‐synthetic dealumination of H‐ZSM‐5 (Figure 7). This is in contrast to trends from da Silva et al.^[8]^ The intermediate time to formation of monocyclic aromatics and the timescale of migration of the hydrocarbon intermediates through the active zeolite surface become equal at the modified sample and contact time of 0.3–0.45 s.

In case of original H‐ZSM‐5 the number of active sites involved in the ethanol conversion to aromatics is higher and the timescale of conversion of intermediate hydrocarbon molecules is lower than the timescale of migration through the catalyst bed. Consequently, the formation of coke and the rate of deactivation are higher. Moreover, for modified H‐ZSM‐5 the limited yield to formation of monocyclic aromatic hydrocarbons (aromatics index (***AI***)) by hydride transfer and cyclization from olefins rises to 70 wt. % of the theoretical maximum.

A summary of the catalytic data reveals a good production of small aromatics with original H‐ZSM‐5. Furthermore, the aromatics yield as well as durability are highly increased after structure and process parameter adaption (see Table 2).

**Table 2:** Summary of material properties and performance of originalH‐ZSM‐5, after post‐synthetic modification and contact time adaption compared to target values from theory.

	Original H‐ZSM‐5	Modified H‐ZSM‐5	At higher contact time	Target value from theory
Si/Al	11	20	20	20–25
(***AI***) [%]	8	23	70	∼100
Coke (10 h ToS) [wt.%]	6.2	3.0	2.6	(lower)
(TOS)_S_ [h]	9	80	123	(longer)

**Figure 8:**
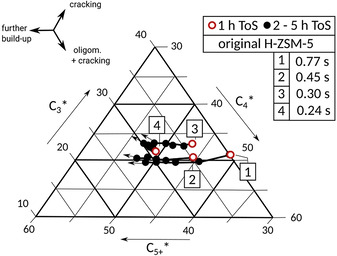
Ternary plot of model‐based formation trends of C_3_, C_4_ and C_5+_ gas‐phase products [wt. %] with ToS [h]: original H‐ZSM‐5 during first 5 h ToS at different contact time (by varying of nitrogen flow).

From a molecular point of view, original H‐ZSM‐5 shows a typical product formation behavior in gas phase. With increasing ToS the probability to form C_5+_ products increases during the first 5 h. Within this product propagation period an increase in contact time (4→1) leads to a tendency of increased C_3_, C_4_ (cracking) and coke production (1 h ToS, see Figure 8).

An increased number of build‐up events becomes probable, which increases the intermediate molar weight of product molecules to blocking coke precursors, which do not contribute to C_5+_ products.

**Figure 9:**
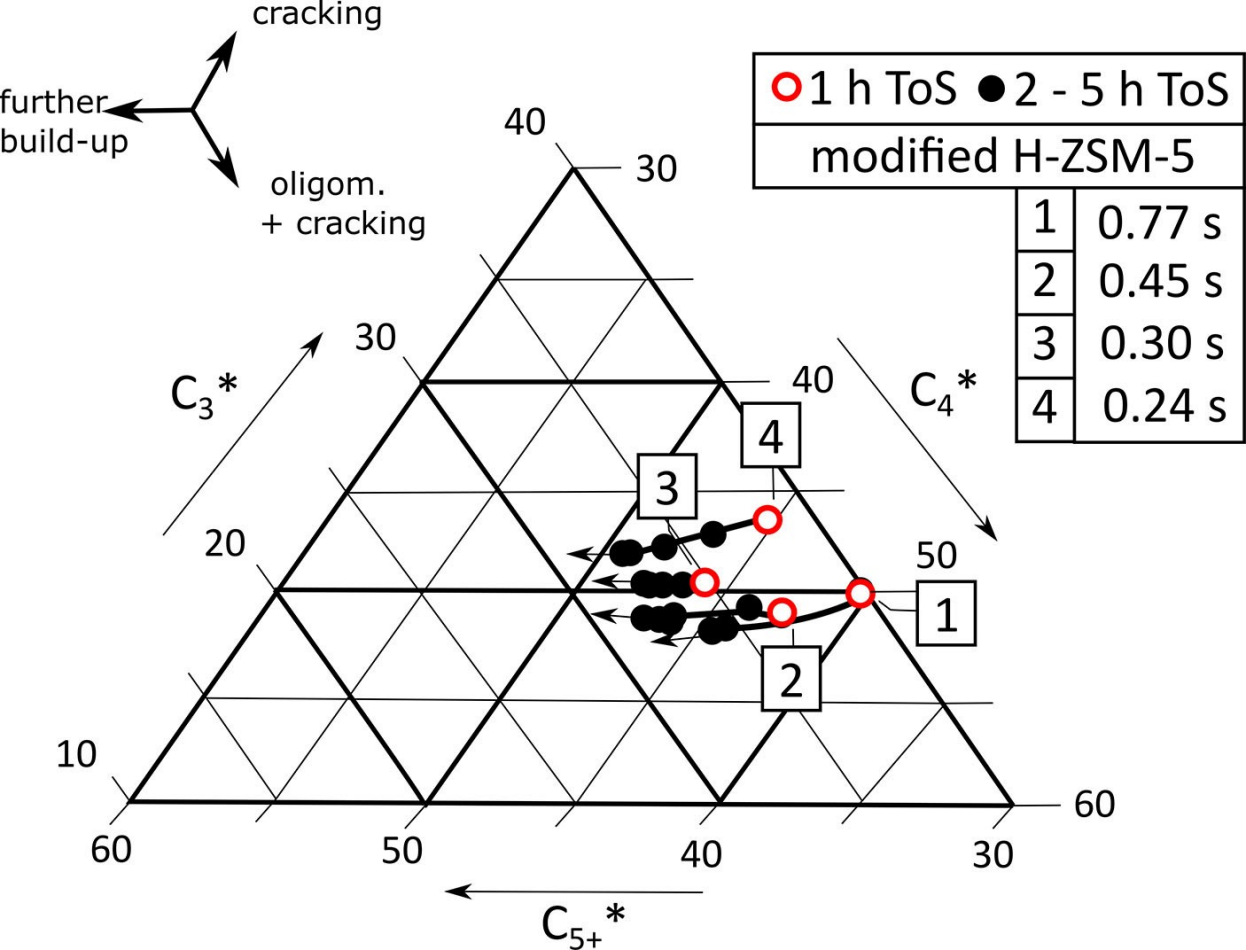
Ternary plot of model‐based formation trends of C_3_, C_4_ and C_5+_ gas‐phase products [wt. %] with ToS [h]: modified H‐ZSM‐5 during first 5 h ToS at different contact time (by varying of nitrogen flow).

A look at the product formation on modified H‐ZSM‐5 in gas phase consisting of C_3_, C_4_ and summarized C_5+_ reveals a trend of a maximum / a plateau of initial C_5+_ formation probability (1 h ToS) at contact time of 0.3 s. At higher contact time (0.77 s) C_3_ and C_4_ (cracking) is more dominant. Therefore, the unusual build‐up and deactivation behavior of modified H‐ZSM‐5 with maximum build‐up and deactivation rate is also observed in C_5+_ formation at a contact time of 0.3 s (see Figure 9).

### III. Mechanistic explanation of the improved durability of the catalyst and the preferred formation of aromatics

**Figure 10:**
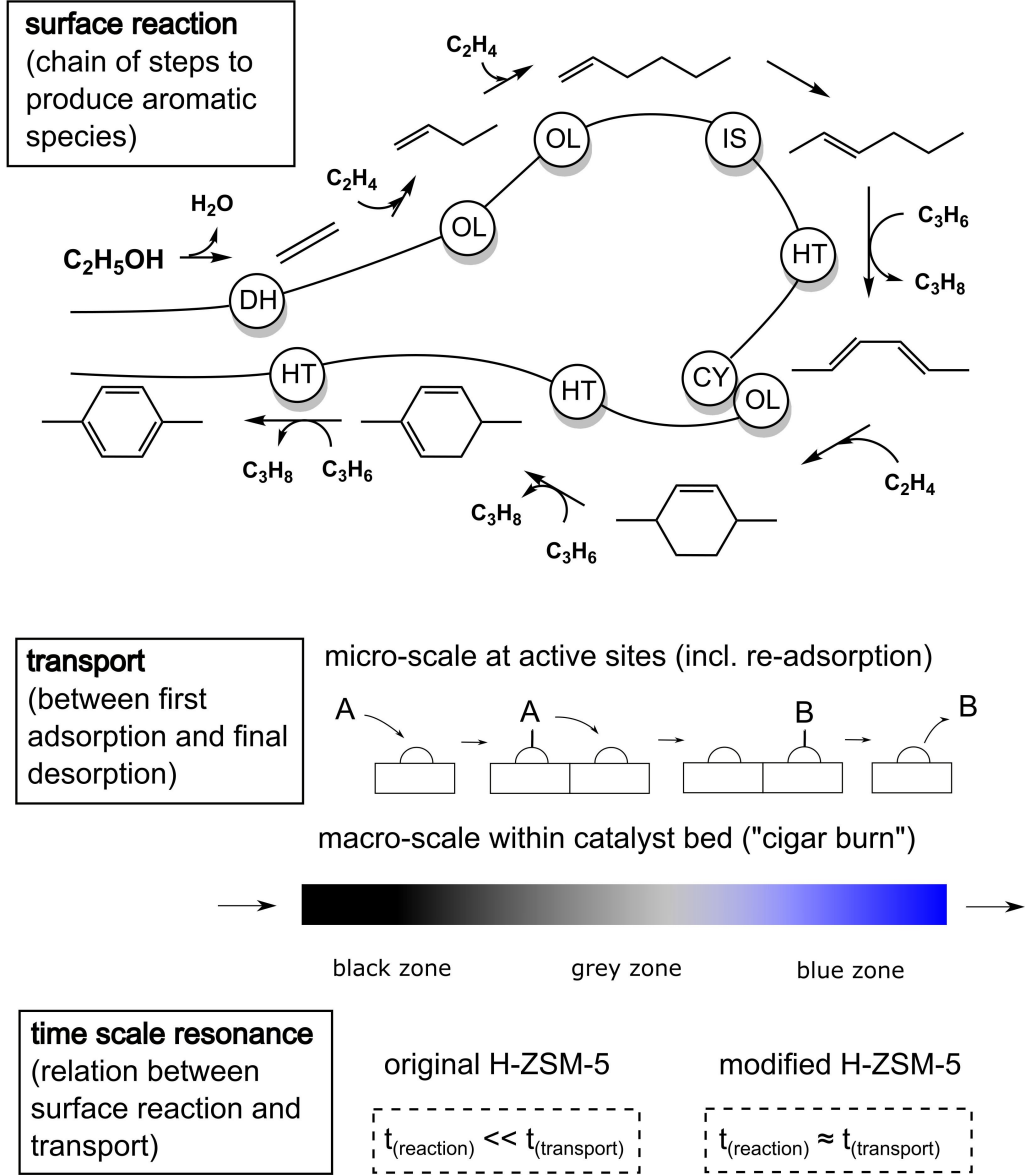
Scheme of harmonized product formation and transport:(top) stepwise formation of aromatics and coke precursors along chain propagation, elementary steps: (DH) dehydration, (OL) oligomerization, (IS) isomerization, (HT) hydride transfer, (CY) cyclization, (CR) cracking; (middle) relation between the reaction at the active sites and the migration through the catalyst surface (micro‐scale) and the catalyst bed (macro‐scale); (bottom) comparison of original H‐ZSM‐5 (conversion too fast) and modified zeolite (conversion timescale near timescale of migration at 0.3–0.45 s).

Surface reaction from ethanol to hydrocarbons and finally coke is considered as a harmonized transport and chemical surface reaction of hydrocarbon intermediates.^[14]^ The number of reaction steps suitable to form aromatics as dominant product has to be in the same time scale as the adsorption, maybe re‐adsorption and migration through the catalyst particle surface and catalyst bed until the final desorption (Figure 10). Based on these premises three model principles can be formulated:

- The probability and speed of each competing elementary reaction step is pre‐determined mainly by microkinetics, i. e. the geometric arrangement of neighboring acid sites (so‐called multiplets), adsorbed intermediary hydrocarbons and temperature, pressure etc. *(static microkinetics)*.
- The statistical number of elementary steps, that may happen from adsorption of first ethanol molecules to final release of reaction products, and the probability and speed of each elementary reaction type determine the dominant product fraction, e. g. small olefins, small aromatics or bigger coke precursors *(dynamic chain length)*.
- The total number of reaction events of each reaction chain is statistically distributed. Short dynamic chains do not exclude coke formation, but probability of coke formation is reduced *(statistical macro‐kinetics)*.

The resulting model of a “statistical chain length mechanism” explains the observed unusual deactivation behavior of modified H‐ZSM‐5 by classical elementary reactions, e. g. hydride transfer, of carbocations:^[14]^

The maximum in deactivation rate within varying contact time on modified H‐ZSM‐5 shows, that aromatics formation is not necessarily an intermediary reaction step to coke precursors as reported by da Silva et al.^[7]^ Meanwhile, higher amounts of coke are not necessarily the reason for an increased catalyst decay. Higher contact time starts to promote the increase of (***AI***) with an unusual low increase in coking, but without direct increase in deactivation.

Increased hydride transfer probability to final aromatization and cyclization by reduced oligomerization determine an overabundant speed to aromatization at modified (dealuminated) H‐ZSM‐5. This becomes more dominant with increased contact time. Higher acid site concentration on the surface of original H‐ZSM‐5 leads to overabundant growth (oligomerization) beyond lower hydride transfer probability. This causes longer statistical reaction event chain lengths and, therefore, a preferred formation of bigger coke precursors, rather than small aromatics.

## Conclusion

Improved efforts in dealumination of a commercial H‐ZSM‐5 are demonstrated by a strategic combination of desilication and dealumination. A stronger framework dealumination as well as pore and bulk cleaning by consecutive washing tend to reduce coke formation during ethanol conversion, increase catalyst durability and increase model‐yields of aromatics (***AI***) up to 23 wt. %. Decrease of space velocity or increase of contact time by reduction of inert gas flow leads to highest durability (9 h→123 h), lowest coke formation after 10 h ToS (6 wt.%→3 wt. %) and highest (***AI***) (8 wt.%→70 wt. %).

Variation of space velocity revealed a resonance phenomenon of time scale between hydrocarbon and coke formation as well as slow transport of hydrocarbons, which results in an unusual maximum in catalyst decay. Finally, the highest yield of aromatics is achieved at high contact time and simultaneously coke formation trend as well as catalyst deactivation are reduced.

The scope of this work is related explicitly to the combined consideration of micro‐scale molecular and surface chemistry as well as macro‐scale reaction engineering concerning the catalyst bed. Especially Schulz et al. for methanol^[15]^ and da Silva et al. for ethanol conversion focus these topics.^[7]^ The distinctive novelty of this research is based on the comprehensive quantification of an unusual catalyst decay, with product and coke formation under space velocity variation. From this a new mechanistic explanation is proposed.

## Experimental Section

### Procedures for dealumination and desilication:

Steaming (St) was carried out in a vertical glass tube reactor with 5 g zeolite (dry) in nitrogen flow with 15 L/h and water with 2 g/h. Temperature was increased with 2 K/min to 400 °C without water flow and held for 12 h under atmospheric pressure.

Basic washing (NaOH) of 15 g (dry) zeolite samples was carried out for 24 h with 0.5 M NaOH (99%) from Grüssing GmbH. Samples are washed with hot water (85–90 °C) afterwards in portions of 10×300 mL.

Acidic washing (HCl) of 15 g (dry) samples was performed over 24 h/96 h with 0.5 M hydrochloric acid (37%) from Sigma Aldrich. Samples were washed as in NaOH.

Each step was followed by drying at 60 °C for 12 h and water vapour saturation over saturated (NH_4_)_2_SO_4_ for reproducible weighting in addition to thermogravimetric analysis (TGA, see below). For complete modification of original H‐ZSM‐5 provided by Clariant (Si/Al=11) a sequence of 100 g of original H‐ZSM‐5 for NaOH (24 h, 0.5 M), 75 g of sample for HCl (96 h, 0.5 M), 10×5 g sample St (400 °C, 12 h) and 15 g of the final sample for HCl (24 h, 0.5 M) treatment was used. During post‐synthetic treatment of original ZSM‐5 with base and acid a subsequent washing with hot, boiling water over a glass filter (Por4) was necessary to remove leached silica and alumina. The treatment with base or acid leads after 24 h to first precipitation at the surface of the round flask. The effect is well‐known as Oswald ripening or re‐crystallization of silica and alumina.^[16]^ The hot solution was transferred to the filter and was separated from mother liquor within 10 min avoiding cooling as good as possible. An electric kettle was used to speed‐up the process of washing.

### Solid‐state and surface characterization

*TPAD*: The acidic properties of the samples were investigated by temperature‐programmed desorption of ammonia (TPAD). All measurements were carried out on a ThermoScientific TPDRO 1100 instrument equipped with a thermal conductivity detector (TCD). Typically, about 300 mg of catalyst sample were used. Before ammonia adsorption, the samples were dried under flowing argon at 550 °C and cooled down to the adsorption temperature of 100 °C. Ammonia adsorption was performed for 30 min. Ammonia desorption was investigated under helium flow by purging the sample chamber for 30 min and subsequent heating up to 700 °C for 1 h with a heating rate of 10 K/min. For calculation of weak (S1) and strong (S2) acidity from TPAD profile a rough division of the integral at 1800 s (417 °C) was used due to a similar TPAD profile of each sample. For calculation of mmol(NH_3_) per gram zeolite from the TCD signal (thermal conductivity detector) a calibration factor was used. (k=7.353347*10^−7^ mmol/(mV*s)) A complex curve fitting with asymmetric Gauss function neither enhanced the accuracy nor changed any trends. For detailed results, please see SI, Figure S1.

*N_2_ physisorption*: All samples were calcined under vacuum. The standard procedure was a calcination at 250 °C for 8 h. After calcination all samples were cooled in liquid nitrogen and physisorption isotherms were collected. (results see SI, Figure S3) The measurements were carried out on a Sorptomatik 1900 from Carlo Erba Instruments and fitted according to BET^[17]^ and BJH theory.^[18]^

*XRD*: X‐ray powder diffraction experiments to investigate the crystallinity of prepared zeolite samples were performed with a SuperNova from Rigaku Oxford Diffraction by accumulation over 10 min with Cu−Kα radiation at 100 K. For calculation of crystallinity by XRD, a modified method of ASTM D5758‐01 was used.^[19]^ Within this standardized method the total area of the reflex group near 24.3° 2θ or the relative heights of the biggest and smallest reflex at the group at about 24° 2θ are used. In accordance to the second method the height of the biggest reflex at 24° 2θ is used for calculation. Related to the first method the absolute height of this reflex is used, but in relation to the reflex at about 8° 2θ, which is not only sensitive to ZSM‐5. As reference the original H‐ZSM‐5 was used and set to 100%. There is an artifact of crystallinity >100%, which occurs if organic templates are inside the zeolite structure (24° 2θ increased) or if silica‐rich crystalline material is removed (8° 2θ decreased).

$$cryst.=\frac{{\left(\frac{max(22\cdots 24^{\circ }2\theta )}{max(7.8\cdots 8.4^{\circ }2\theta )}\right)}_{zeolite\;sample}}{{\left(\frac{max(22\cdots 24^{\circ }2\theta )}{max(7.8\cdots 8.4^{\circ }2\theta )}\right)}_{original\;ZSM-5}}\cdot 100\%$$

For calculation a refinement of the diffractogram was done by a curve fitting. A device and angle dependent underground signal was subtracted by a degressive exponential function. The sample preparation with a nylon loop and a viscous oil showed a almost quadratic underground signal with migrating maximum at about 20 to 30° 2θ. The correction was done by 3 different quadratic functions, which were subtracted prior to calculation of the crystallinity. Processed powder diffraction patterns are provided in Figure S3 within the SI.

*TGA*: Thermogravimetric analysis was performed with a TG50 from Mettler Toledo under nitrogen flow (200 mL/min) to evaluate dry mass of each sample and under synthetic air (200 mL/min) for coke mass determination after catalytic tests. Temperature program consists of a ramp between 35–850 °C with a rate of 10 K/min each run.

*ICP‐OES*: Elementary analysis was performed by dissolution of zeolite powder by microwave assisted solvation with a mixture of nitric, hydrochloric and hydrofluoric acid. Quenching with boric acid was followed by quantitative determination of silicon, aluminum and sodium by inductive‐coupled plasma combined with optical emission spectroscopy. For analysis of solutions an Optima 2000 DV by PerkinElmer was calibrated to Si at 212.4 nm, Al at 396.2 nm and Na at 589.0 nm. Calibration was done by defined solutions of 1000 mg/L standards by PerkinElmer and deionized water.

*Particle size analysis*: Dynamic laser scattering was used to determine particle size distribution with a Bettersizer S3 Plus by 3P Instruments. Wet dispersion procedure was performed with 2000 rpm stirring and 200 W (26 kHz) ultrasonic for 2 min. Data acquisition and proceeding are in accordance to ISO 13320 : 2009 with calculation of particle size distribution from scattering data by Mie theory.^[20]^

*Catalytic measurement*: For the catalytic testing zeolite powder materials were pressed at 1.5–2 MPa, crushed and sieved in a range of 315–400 μm. The conversion was carried out in a steel tubular fixed‐bed reactor. For each run 500 mg catalyst sample (dry mass) was used. Reaction temperature for catalytic testing was 350 °C controlled by a thermocouple directed heating. Ethanol (2.5 g/h) and nitrogen carrier gas (15 L/h) were fed into the reactor via heated pipes (WHSV of 5 h^−1^). During 10 h ToS and 2.5 bar (absolute pressure) condensable products were trapped in a liquid separator. In all cases, a mass balance was defined including yield of coke, liquid condensable and gas‐phase products with regard to ethene. The resulting gaseous and liquid reaction mixtures were analysed by gas chromatography using an HP 5890 Series‐II GC System equipped with an FID (flame ionization detector) and a non‐polar 100 m HP‐1 column. Pre‐tests showed no or only traces of ethanol or diethyl ether at above 300 °C. Prior to catalytic testing all samples were dried at 120 °C for 1 h in nitrogen (5 L/h) following a calcination step (rate 3 K/min) at 450 °C for 0.5 h. A detailed test plan is displayed in Table 3.

**Table 3:** Overview of flow parameters for experiments under variation of contact time, catalyst mass=500 mg (dry), 20 PSIG (1.4 bar) overpressure, WHSV=5 h^−1^.

Run	Nitrogen [L/h]	Ethanol [g/h]	Ethanol fraction in feed [Vol%]	Contact time [s]
1	5.0	2.5	21.0	0.77
2	10.0	2.5	11.7	0.45
3^[a]^	15.0	2.5	8.1	0.30
4	20.0	2.5	6.2	0.24

[a] Run 3 was the standard setting for catalytic tests of original and modified H‐ZSM‐5 within the initial comparison of both materials.

During an increase of nitrogen flow the contact time and ethanol partial pressure in the feed drop simultaneously. Although, reduced ethanol concentration may also cause changes in coking and product formation behavior, the setup was chosen, because:

- Water from dehydration act as inert gases, because there is only negligible competition of adsorption at acid sites compared to olefinic hydrocarbons, concluded by van der Borght et al.^[21]^
- From perspective of a reduced ethanol partial pressure by increasing nitrogen flow, there is a reduced probability to adsorption / re‐adsorption and therefore a reduction of the average reaction chain length from ethanol to the major product fraction. Consequently, for both considerations, ethanol partial pressure and contact time, coke precursors and aromatics should be reduced.
- From the perspective of different power laws of interconverting chemical reactions, there is a strong tendency to effective power 1 for small aromatics because of diffusion limitations.^[22]^ A change in nitrogen flow should not affect the flow regimes too much, but the contact time.
- Discussion for speed‐up of power 2 molecular reactions as discussed for bimolecular hydride transfer and cyclization and observed maxima in coke and aromatics formation is complementary to the discussion of the reaction chain length.

*Product analysis and mass balance*: Product analysis was standardized by DHA method (detailed hydrocarbon analysis) for determination of hydrocarbons including response factor analysis with HP 5890 Series‐II GC System from Agilent. Mass balance was calculated from gas and liquid phase analysis by GC and balancing of liquid phase products and coke analysis by TGA. Data analysis was done for each individual compound and summarized in fractions such as olefins, paraffins and aromatics. Chemical composition of coke was neglected due to its low amount below 1 wt. % of full mass balance. Compound determination in gas phase was compared to results from gas chromatography and mass spectrometry (GC/MS) with a Clarus 680 GC and a Clarus SQ 8 S MS from PerkinElmer with an extended DHA method. Further details to testing parameters and calculations are collected at the SI, Table S2. Further details concerning mass balance and deactivation plot model are listed at the end of the supplementary information. (Supplementary Experimental Section).

## Conflict of interest

The authors declare no conflict of interest.

## Supporting information

As a service to our authors and readers, this journal provides supporting information supplied by the authors. Such materials are peer reviewed and may be re‐organized for online delivery, but are not copy‐edited or typeset. Technical support issues arising from supporting information (other than missing files) should be addressed to the authors.

Supplementary
