# Supplementary material for: Ethanol to Aromatics on Modified H‐ZSM‐5 Part II: An Unexpected Low Coking
Source: Chem Asian J. 2020 Oct 20;15(22):3878–85. doi: 10.1002/asia.202000961 (PMC7756217; doi:10.1002/asia.202000961)
Supplement: Supplementary file 1 — Supplementary [file ASIA-15-3878-s001.pdf]

CHEMISTRY
AN ASIAN JOURNAL

Supporting Information

Ethanol to Aromatics on Modified H-ZSM-5 Part II: An Unexpected Low Coking

Markus Seifert, Mathias S. Marschall, Torsten Gille, Clemens Jonscher, Philipp Royla, Oliver Busse, Wladimir Reschetilowski, and Jan J. Weigand*© 2020 The Authors. Chemistry - An Asian Journal published by Wiley-VCH GmbH. This is an open access article under the terms of the Creative Commons Attribution License, which permits use, distribution and reproduction in any medium, provided the original work is properly cited.

Multiplet concept adapted to Kobozev et al.

Table S1. Results of a geometric consideration of homogeneous and statistical distribution of framework aluminum in ZSM-5, EFAL not considered: correlation between Si/Al ratio and framework acid site multiplets in adaption to model of isolated ensembles by Nikolaj I. Kobozev [1]

Si/Al ratio	Al per unit cell	Al per pore intersection	Most common acid site multiplet per pore intersection
1.91	33 [a]	8	--
6.4 – 7.0	13 – 12	3	triplet
9.7 – 11	9 – 8	2	doublet<br>(+ triplets)
18 – 23	5 – 4	1	singlet<br>(+ doublets)
31 - 47	3 – 2	0.5	singlet
95	1	0.25	singlet (isolated pore intersection)

[a] Estimation of aluminum distribution according to Löwenstein's rule, i.e. no [AlO_4] tetrahedron is directly connected to another one. [2]

As the key reactions to coking and aromatics formation within hydrocarbon pool of methanol and ethanol conversion the cyclization and hydride transfer are accepted. Both mechanisms benefit from neighboring Brønsted acid sites, i.e. for the model: Pre-coordination and re-adsorption is facilitated by site multiplets consisting of two sites (doublets). On the other hand, overabundant sites increase the probability of corresponding coke formation through pore channels beyond more than one site multiplet. A fast formation of small aromatics and moderate formation of coke needs a few isolated acid site doublets, but not too much to avoid a multiplet based hydrocarbon growth. (Si/Al = 18 - 23)

Including amounts of EFAL and slight inhomogeneities in aluminum distribution [3] into consideration, the most promising Si/Al ratio might increase slightly. (Si/Al ≈ 20 - 25)

Table S2. Temperature program for gas and liquid phase analysis related to DHA standard method. [4]

**Gas phase analysis**					
Ramp [K/min]	Start [°C]	End [°C]	Dwell [min]	He flow [mL]	Pressure [kPa]
0	35	35	13.0	1.2	254
10	35	45	15.0	1.2	254
1	45	55	0.0	1.2	254
**Liquid phase analysis**					
Ramp [K/min]	Start [°C]	End [°C]	Dwell [min]	He flow [mL]	Pressure [kPa]
0	35	35	13.0	1.2	254
10	35	45	15.0	1.2	254
1	45	60	10.0	1.2	254
1	60	200	30.0	1.2	254

Calculations to develop a full mass balance in hydrocarbon conversion

Calculation concerning feed:

feed and workload *("tot" = total)*

$$\dot{m}_{EtOH,l} = \frac{\Delta m_{tot}}{\Delta(ToS)_{tot}} = \frac{\rho \cdot \Delta V_{tot}}{\Delta(ToS)_{tot}}$$

$$with\ \Delta(ToS)_{tot} = 10\ h\ \ and\ \rho = 0.7893\ \frac{g}{mL}$$

$$with\ WHSV = \frac{\dot{m}_{EtOH,l}}{m_{cat.}} = 5\ h^{-1}\ \ and\ \ m_{cat.} = 0.5\ g$$

total amount of ethanol

$$\Delta m_{EtOH,tot} = \dot{m}_{EtOH,l} \cdot \Delta(ToS)_{tot}$$

$$with\ \dot{m}_{EtOH,l} = 2.5\ \frac{g}{h}$$

total amount of ethene

$$\Delta m_{Ethene,tot} = \Delta m_{EtOH,tot} \cdot \frac{M_{Ethene}}{M_{EtOH}}$$

$$with\ M_{Ethene} = 28.05 \frac{g}{mol}\ and\ \ M_{EtOH} = 46.07 \frac{g}{mol}$$

Calculation concerning coke:

total amount of coke *("cat" = catalyst)*

$$m_{coke,tot} = \omega_{TGA} \cdot m_{cat}$$

Calculation concerning organic liquid phase:

total amount of organic liquid phase

$$m_{liq,tot} = \Delta m_{balance}$$

assumption for aromatics *("aro" = aromatics)*

$$m_{liq,aro} \approx m_{liq,tot}$$

Calculation concerning organic gas phase:

total amount of organic gas phase

$$m_{gas,tot} = \Delta m_{Ethene,tot} - m_{coke,tot} - m_{liq,tot}$$

fractions within organic gas phase

$$m_{gas,i} = \frac{A_{GC,i} \cdot RF_i}{\sum_j A_{GC,j} \cdot RF_j} \cdot m_{gas,tot}$$

assumption for aromatics

$$m_{tot,aro} = m_{liq,aro} + m_{gas,aro}$$

$$with\ m_{gas,aro} = m_{gas,benzene} + m_{gas,toluene}$$

The total amount of small condensable aromatics is defined as the sum of liquid phase and aromatics from gas phase (toluene and benzene). For comparison a theoretical limit is estimated for production of aromatics from ethanol. The following side conditions are included in this estimation:

(i) Primary reaction of ethanol leads to ethene and water, i.e. there is a mass loss due to water removal.

(ii) There is a restriction of formation of aromatics by cyclization and hydride transfer from olefins. About an inevitable production of paraffins in molar ratio to aromatics 3:1 is reported in case there is no production of molecular hydrogen. [5,6] A mass loss in paraffin production has to be included.

(iii) Furthermore, the activity of ethene within carbocation based reactions is very limited. Small olefins such as propene or butenes are more favorable hydride donor or acceptor molecules. [7]

An overview of model based total formula of conversion reaction and corresponding theoretical limits in production of aromatics is added in Table S3.

Table S3. Summarized total formula and corresponding theoretical limits in production of aromatics (in %) from ethene and ethanol conversion.

$M(C_8)$ = 106.2 g/mol	From ethene $M(C_2H_4)$ = 28.05 g/mol	From ethanol $M(C_2H_5OH)$ = 18.02 g/mol
**propene** as hydride acceptor	44.5 %	27.1 %
total formula	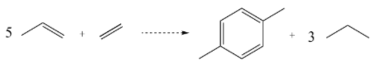	
butene as hydride acceptor	37.9 %	23.1 %
total formula	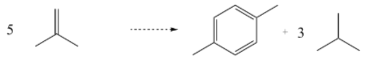	

For estimation of the efficiency of zeolite samples to produce aromatics, a new parameter, the aromatics index (***AI***) is defined, which quantifies the fraction of aromatics in relation to the theoretical limit calculated from propene (see Table S4).

$$(\boldsymbol{AI}) = \frac{\boldsymbol{carbon\ yield\ (aromatics)_{(ToS=10\ h)}}}{\boldsymbol{theoretical\ limit\ (aromatics)_{(ToS=10\ h)}}} \cdot \mathbf{100}\%$$

The origin of ethane from ethene by hydride transfer is not excluded, but the probability for C3 and C4 seem to be much higher as reported by Pinard et al. [8] Ethylene is less active in acid catalyzed hydride transfer reactions compared to propene. Consequently, propene as preferred hydride acceptor (intermediate of C2, C3 and C4) is a first rough guess for aromatics index (***AI***) estimation. From experimental data the total molar fractions in gas phase [%] and the ratio of alkane to olefin for original and modified H-ZSM-5 after 2 h ToS and standard test conditions (350°C etc.) are summarized in Table S4. Beside short-chain alkane formation from cracking, an increased ratio alkane:olefin from C2 (~0.1) to C4 (~1.5) confirms the expected trend from literature.

Table S4. Experimental data of the total molar fractions in gas phase [%] and the ratio of alkane to olefin for original H-ZSM-5 after 2 h ToS and standard test conditions (350°C, 2.5 bar, WHSV = 5 h^{-1}).

**Selected gas-phase fractions of original H-ZSM-5**			
Fraction	Alkane [mol%]	Olefin [mol%]	Ratio alkane : olefin
C2	0.61	5,63	0.11
C3	2.49	4.66	0.53
C4	5,74	3.87	1.48
**Selected gas-phase fractions of modified H-ZSM-5**			
Fraction	Alkane [mol%]	Olefin [mol%]	Ratio alkane : olefin
C2	0.54	5.23	0.10
C3	2.01	4.39	0.46
C4	6.36	3.98	1.60

Deactivation plot of ethene conversion in ETH process adapted to Yuan et al. and Bremer et al.

From a fundamental description of catalyst grain deactivation [7] a simple 1st order kinetic model is used to fit gas phase C2 fraction to the ToS. Similar models are proposed recently for methanol conversion depending of ToS in MTH process. [9] In analogy to this model the C2 fraction is interpreted as unconverted ethene, i.e. the model conversion is defined as ($C2_{max}$ - C2).

Differential equation:

$$\frac{d[C_2]}{d(ToS)} = \underbrace{k' \cdot WHSV^n \cdot \alpha}_{k} \cdot [C_2] \cdot ([C_2]_{max} - [C_2])$$

Micro-kinetic solution (1 cell):

$$[C_2]_i = \frac{[C_2]_{max}}{1 + \left(\frac{[C_2]_{max}}{[C_2]_{i-1}} - 1\right) \cdot exp\left(-k \cdot [C_2]_{max} \cdot (ToS)\right)}$$

The micro-kinetic model was transferred to a plug-flow reactor model (PFR) within cell model. [10] The description of the catalyst bed coking as a 'cigar burning' or an absorber column simplifies the description to a migrating reactive zone within the catalyst bed during ToS. Further simplification revealed $C2_{max} \approx 1.0$. With ongoing deactivation, the dehydration remains very fast, whereas the secondary conversion of ethene tends to stop completely.
Macro-kinetic solution (PFR): (N cells)

$$[C_2] = \frac{[C_2]_{max}}{1 + \left(\frac{[C_2]_{max}}{[C_2]_0} - 1\right) \cdot exp\left(-N \cdot k \cdot [C_2]_{max} \cdot (ToS)\right)}$$

Other more complex models are tested without any improvement of the fit (2nd order PFR, 3rd order PFR, 1st order laminar reactor). Therefore, the cigar burning type 1st order PFR model was used within this work.
The model deactivation rate *(N*k)* as well as the initial conversion of C2 (1-$[C_2]_0$) as build-up activity characterize the deactivation behavior of a zeolite material. The sigmoid-type shape of the function facilitates the calculation of a deactivation parameter $(ToS)_S$ at its turning point, which describes the ToS at 50% deactivation for $C2_{max} \approx 1.0$.

$$(ToS)_S = \frac{1}{N \cdot k \cdot [C_2]_{max}} \cdot ln\left(\frac{[C_2]_{max}}{[C_2]_0} - 1\right) \approx \frac{1}{N \cdot k} \cdot ln\left(\frac{1}{[C_2]_0} - 1\right)$$

Considering small olefins, such as propene or butenes, as highly reactive intermediates [10] to the key reaction step for hydride transfer as main coke forming process [12], changes in gas-phase product distribution of C_3, C_4 and C_{5+} with ToS are summarized in a model-based ternary plot.
Product formation with ToS leads to an increase of C_2 fraction (ethene + ethane) and a complex behavior of other product molecules C_{3+}. From the assumption, that H-ZSM-5 leads to complete dehydration of ethanol at 350°C reaction temperature, all products C_{3+} may be considered as follow-up products (see Figure 3). Consequently, the standardization of a conversion of C_2 leads to a second conversion parameter (1-$[C_2]$) after first ethanol conversion. By standardization of the summarized fractions of C_3, C_4 and C_{5+} hydrocarbons in gas phase, trends become visible, which are independent from the general trend of an C_2 increase. Because of the use of three fractions normalized to 100 % by a standardization to secondary C_2 conversion, C_3*, C_4*, C_{5+}*, a triangular plot is used for graphical illustration.

Supplementary Results

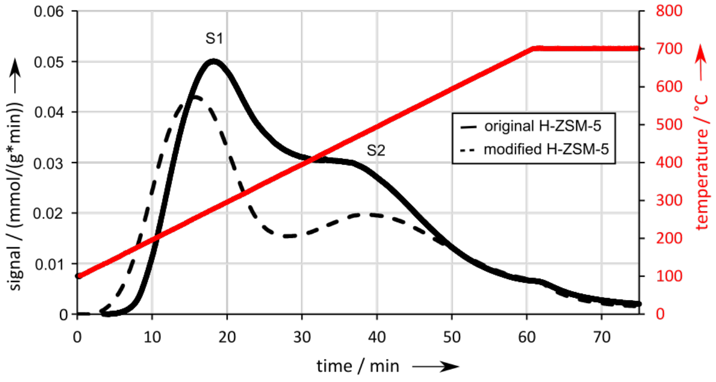

Figure S1. Profiles of temperature-programmed desorption of ammonia (TPAD) until 700°C for original H-ZSM-5 and modified H-ZSM-5.

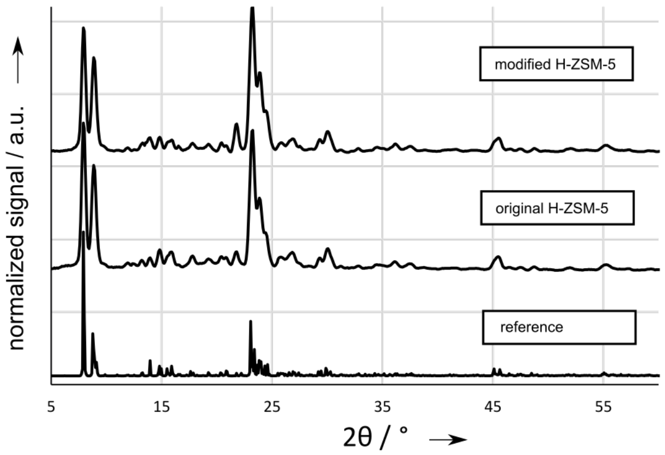

Figure S2. Processed powder diffraction patterns of original H-ZSM-5, modified H-ZSM-5 and reference of the IZA database. [13]

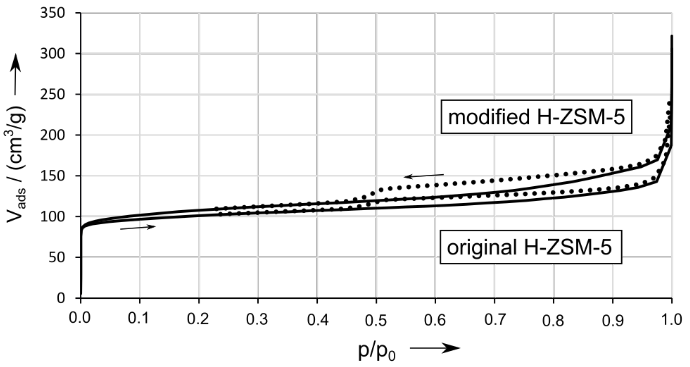

Figure S3. Isotherms for physisorption of nitrogen of original H-ZSM-5 and modified H-ZSM-5.

References

[1] 1 N. M. Emanuel, *Russ. Chem. Rev.* **1974**, *43(5)*, 317-362.

[2] 5 W. Löwenstein, *Am. Mineral.* **1954**, *39*, 92-96.

[3] D. E. Perea, I. Arslan, J. Liu, Z. Ristanovic, L. Kovarik, B. W. Arey, J. A. Lercher, S. R. Bare, B. M. Weckhuysen, *Nature Comm.* **2015**, *6*, 7589.

[4] AC DHA Analyzer, *Detailed Hydrocarbon Analysis by High Resolution Capillary Gas Chromatography*, 1994, AC Analytical Controls Inc., Bensalem, Pennsylvania 19020 USA

[5] F. F. Madeira, N. S. Gnep, P. Magnoux, H. Vezin, S. Maury, N. Cadran, *Chem. Eng. J.* **2010**, *161*, 403-408.

[6] F. F. Maderia, K. B. Tayeb, L. Pinard, H. Vezin, S. Maury, N. Cardran, *Appl. Catal. A* **2012**, *443*, 171-180.

[7] H. Bremer, N. Michael, W. Reschetilowski, K.-P. Wendlandt, *Wiss. Z. TH Leuna-Merseburg* **1983**, *25(2)*, 205-213.

[8] S. Hamieh, C. Canaff, K. B. Tayeb, M. Tarighi, S. Maury, H. Vezin, Y Pouilloux, L. Pinard, *Eur. Phys. J. Special Topics* **2015**, *224*, 1817-1830.

[9] X. Yuan, H. Li, M. Ye, Z. Liu, *Chem. Eng. J.* **2017**, *329*, 35-44.

[10] H. Schulz, M. Wei, *Top. Catal.* **2014**, *57*, 683-692.

[11] S. Hamieh, C. Canaff, K. B. Tayeb, M. Tarighi, S. Maury, H. Vezin, Y Pouilloux, L. Pinard, *Eur. Phys. J. Special Topics* **2015**, *224*, 1817-1830.

[12] S. Müller, Y. Liu, F. M. Kirchberger, M. Tonigold, M. Sanchez-Sanchez, J. A. Lercher, *J. Am. Chem. Soc.* **2016**, *138*, 15994-16003.

[13] C. Baerlocher, L. B. McCusker, Database of Zeolite Structures: http://www.iza-structure.org/databases/
